# Live cell imaging of macrophage/bacterium interaction demonstrates cell lysis induced by *Corynebacterium diphtheriae* and *Corynebacterium ulcerans*

**DOI:** 10.1186/s13104-019-4733-y

**Published:** 2019-10-25

**Authors:** Dulanthi Weerasekera, Jonas Hahn, Martin Herrmann, Andreas Burkovski

**Affiliations:** 1 Friedrich-Alexander-Universität Erlangen-Nürnberg, Staudtstr. 5, 91058 Erlangen, Germany; 2 Friedrich-Alexander-Universität Erlangen-Nürnberg, Universitätsklinikum Erlangen, Erlangen, Germany

**Keywords:** Cytotoxicity, Diphtheria, Live cell imaging, Phagocytes, THP-1, Time lapse fluorescence microscopy

## Abstract

**Objectives:**

In frame of a study to characterize the interaction of human macrophage-like cells with pathogenic corynebacteria, *Corynebacterium diphtheriae* and *Corynebacterium ulcerans*, live cell imaging experiments were carried out and time lapse fluorescence microscopy videos were generated, which are presented here.

**Data description:**

The time lapse fluorescence microscopy data revealed new insights in the interaction of corynebacteria with human macrophage-like THP-1 cells. In contrast to uninfected cells and infections with non-pathogenic *C. glutamicum* used as a control, pathogenic *C. diphtheriae* and *C. ulcerans* showed highly detrimental effects towards human cells and induction of cell death of macrophages.

## Objective

Within the actinobacteria (high G+C DNA content Gram-positives) the genus *Corynebacterium* forms together with the genera *Mycobacterium*, *Nocardia* and *Rhodococcus* the CMNR group, which is characterized by a complex, mycolic acid-containing cell wall structure [1]. At the time of writing, 132 species and 11 subspecies were assigned to the genus [2], with more than half of these isolated from animal and human sources or clinical material [3]. The most prominent member of the genus is *Corynebacterium diphtheriae*, which forms together with *Corynebacterium ulcerans* and *Corynebacterium pseudotuberculosis* the group of toxigenic corynebacteria [4], based on their common characteristic that they can produce diphtheria toxin (DT), a potent exotoxin, after being lysogenized by *tox* gene-carrying corynephages [5]. DT is responsible for the high fatality rate of diphtheria with an overall death toll of 5 to 10% and a fatality rate up to 20% among children younger than five and in unvaccinated or not sufficiently protected individuals [6]. Interestingly, *C. diphtheriae* and *C. ulcerans* strains are not only able to adhere to and invade epithelial cells, but may also persist inside macrophages after being taken up by these phagocytes [7]. Recent publications showed that *C. diphtheriae* as well as *C. ulcerans* may interfere with phagolysosome maturation in murine and human macrophages after phagocytosis [8–11]. Most recently, a study combining of fluorescence microscopy, cytotoxicity assays and fluorescence-activated cell sorting revealed that these pathogenic corynebacteria induce necroptosis in human phagocytic cell lines [12]. It was suggested that survival in macrophages and subsequent necrotic lysis of cells may be mechanisms for dissemination of *C. diphtheriae* and *C. ulcerans* within the host and support colonization of host tissues far distant from the infection site. Within this context, a collection of time lapse fluorescence microscopy videos was taken, which are presented here.

## Data description

### Live cell imaging

THP-1 human monocytic cells [13] were cultured at 37 °C in 10% fetal calf serum (FCS; Life Technologies, Carlsbad, CA, USA) supplemented Roswell Park Memorial Institute (RPMI) medium 1640 (Thermo Fisher Scientific, Waltham, MA, USA) containing 100 U ml^−1^ penicillin and streptomycin, respectively. For live cell imaging assays, cells were seeded in a density of 1.2 × 10^5^ cells on 8 wells sterile glass bottom µ-slides (Thermo Fisher Scientific, Waltham, MA, USA) and differentiated by addition of 10 ng ml^−1^ phorbol 12-myristate 13-acetate (PMA; Sigma, Darmstadt, Germany) 24 h prior to infection. The cells were washed two times with phosphate-buffered saline (PBS; B.Braun, Melsungen, Germany) to remove non-adherent cells and fresh medium was added. Cells were incubated at 37 °C under humified atmosphere and 5% CO_2_ at least 20 min prior to infection with bacteria. Staining was carried out using 0.1 µg ml^−1^ Hoechst 33342 (Thermo Fisher Scientific, Waltham, MA, USA) and 1 µg ml^−1^ propidium iodide (Invitrogen, Carlsbad, CA, USA) in PBS. For infection, overnight cultures of green fluorescent protein (GFP)-expressing corynebacteria [12] grown in kanamycin-containing Heart infusion (HI) medium (Becton–Dickinson, Sparks, MD, USA) were inoculated to an OD_600_ of 0.1 in fresh medium, harvested at the beginning of the exponential growth phase (OD_600_ approx. 0.4 to 0.6) and used to infect macrophages at an MOI of 25. Micrographs were taken using a BZ-X710 microscope (Keyence, Neu-Isenburg, Germany) and the corresponding the BZ-X710 software package (Keyence, Neu-Isenburg, Germany).

### Time lapse fluorescence microscopy

From the micrographs taken every 15 min over a time period of 20 h, time lapse videos were produced using the BZ-X710 software (Keyence, Neu-Isenburg, Germany) (Table 1). Bacteria are stained in green due to GFP expression, nuclei appear in blue due to DNA staining by Hoechst 33342 (Thermo Fisher Scientific, Waltham, MA, USA) and dead cells with defect membrane barrier are stained red by propidium iodide (Invitrogen, Carlsbad CA, USA).

**Table 1:** Overview of data files/data sets

Label	Name of data file/data set	File types (file extension)	Data repository and identifier (DOI or accession number)
Data set 1 [14]	uninfected cells.mp4	Media file (mp4)	10.6084/m9.figshare.9878708
Data set 2 [15]	C. glutamicum-macrophage interaction.mp4	Media file (mp4)	10.6084/m9.figshare.9878687
Data set 3 [16]	C. ulcerans-macrophage interaction.mp4	Media file (mp4)	10.6084/m9.figshare.9878681
Data set 4 [17]	C. diphtheriae-macrophage interaction.mp4	Media file (mp4)	10.6084/m9.figshare.9810536

Data sets 1 to 4 (Table 1) show the behavior of uninfected cells and cells infected with nonpathogenic *C. glutamicum* ATCC13032 as well as pathogenic *C. diphtheriae* HC04 and *C. ulcerans* 809, respectively. The uninfected cells revealed only a small amount of propidium iodide-stained dead cells over the incubation period of 20 h (15 ± 4% dead cell). Infection with nonpathogenic *C. glutamicum* ATCC13032 showed a slightly increased number of dead cells (25 ± 5% dead macrophages), while propagation of bacteria was poor. In contrast, infection with *C. diphtheriae* resulted in strong induction of cell death (75 ± 5.6% dead cells). Strong bacterial growth and formation of micro-colonies in the medium was detectable in this case. Also in case of *C. ulcerans* strong detrimental effects were observed with 85 ± 12% dead phagocytes after 20 h of infection. Compared to *C. diphtheriae*, enhanced phagocytosis of bacteria and bacterial growth within phagocytes was observed [14–17].

## Limitations

The data sets presented here support and extend results on the interaction of *Corynebacterium* species; i.e. *C. diphtheriae*, *C. glutamicum* and *C. ulcerans*, with different phagocytic cells published previously [8–12]. Limitations of the data sets are (i) the use of only one strain per species applied, (ii) the analysis of only one human macrophage-like cell line and (iii) the use of only one MOI in the infection experiments.

## Data Availability

The data described can be freely and openly accessed on https://figshare.com/. Please see Table 1 and reference list [14–17] for details and direct links to the data.

## Abbreviations

DT: diphtheria toxin
FCS: fetal calf serum
GFP: green fluorescent protein
MOI: multiplicity of infection
OD_600_: optical density at 600 nm wavelength
PBS: phosphate-buffered saline
PMA: phorbol 12-myristate 13-acetate
RPMI: Roswell Park Memorial Institute
